# Self-assembly of ordered graphene nanodot arrays

**DOI:** 10.1038/s41467-017-00042-4

**Published:** 2017-06-29

**Authors:** Luca Camilli, Jakob H. Jørgensen, Jerry Tersoff, Adam C. Stoot, Richard Balog, Andrew Cassidy, Jerzy T. Sadowski, Peter Bøggild, Liv Hornekær

**Affiliations:** 1 Center for Nanostructured Graphene, DTU Nanotech, Technical University of Denmark, Kongens Lyngby, DK-2800 Denmark; 2 Department of Physics and Astronomy and Interdisciplinary Nanoscience Center iNANO, Aarhus University, Aarhus C, 8000 Denmark; 3 IBM Research Division, T.J. Watson Research Center, Yorktown Heights, New York, New York 10598 USA; 4 Center for Functional Nanomaterials, Brookhaven National Laboratory, Upton, New York 11973 USA

## Abstract

The ability to fabricate nanoscale domains of uniform size in two-dimensional materials could potentially enable new applications in nanoelectronics and the development of innovative metamaterials. However, achieving even minimal control over the growth of two-dimensional lateral heterostructures at such extreme dimensions has proven exceptionally challenging. Here we show the spontaneous formation of ordered arrays of graphene nano-domains (dots), epitaxially embedded in a two-dimensional boron–carbon–nitrogen alloy. These dots exhibit a strikingly uniform size of 1.6 ± 0.2 nm and strong ordering, and the array periodicity can be tuned by adjusting the growth conditions. We explain this behaviour with a model incorporating dot-boundary energy, a moiré-modulated substrate interaction and a long-range repulsion between dots. This new two-dimensional material, which theory predicts to be an ordered composite of uniform-size semiconducting graphene quantum dots laterally integrated within a larger-bandgap matrix, holds promise for novel electronic and optoelectronic properties, with a variety of potential device applications.

## Abstract (web summary)

The nanoscale patterning of two-dimensional materials offers the possibility of novel optoelectronic properties; however, it remains challenging. Here, Camilli et al. show the self-assembly of large arrays of highly-uniform graphene dots imbedded in a BCN matrix, enabling novel devices.

## Introduction

Structures approaching the nanometre scale hold great interest, both for basic science and for potential applications in advanced technology. However, controlled fabrication at this scale represents an extreme challenge. Many applications such as quantum dots and metamaterials, moreover, require nanoscale heterostructures integrated within a solid matrix. Intriguing progress has been made using misfit stress to drive self-assembly of nanostructures on surfaces during heteroepitaxy^1, 2^. However, there has been only limited success in achieving uniform size or extremely small dimensions.

The advent of two-dimensional (2D) materials^3^ opens up new opportunities and challenges. Most notably, confining lower dimensional domains within a 2D heterostructure could potentially lead to exciting and useful quantum effects. In particular, 2D lateral heterostructures formed by graphene and hexagonal boron nitride have recently been the subject of intense research^4–10^ owing to the similar lattice parameters but complementary electronic properties of their individual components. Heterostructures of different 2D semiconducting transition metal dichalcogenides are also of great technological interest^11, 12^. However, a route towards fabrication of 2D lateral heterostructures at extreme nanoscale dimensions is still unavailable.

Here we report the self-assembly and self-ordering of 0D graphene dots of highly regular size, 1.6 ± 0.2 nm in diameter, epitaxially embedded within a 2D boron-carbon-nitrogen (BCN) alloy. Using a combination of in situ microscopy, diffraction and spectroscopy techniques, we characterise the monolayer BCN grown on an Ir(111) substrate under different growth conditions. With an increasing C concentration, graphene dots appear in the BCN layer and self-organise into ordered hexagonal arrays. The arrays become denser when we increase the C to BN ratio during the growth process. We show that the observed uniform dot size can be explained by a model describing the competition between the graphene–BCN boundary energy and moiré-type interactions with the substrate, with the long-range organisation provided by the repulsion^13, 14^ between the dots. Furthermore, theory predicts graphene nanodots of the observed size to be semiconducting quantum dots^15, 16^ and the BCN matrix to possess a larger bandgap^17^.

## Results

### STM investigation

**Fig. 1:**
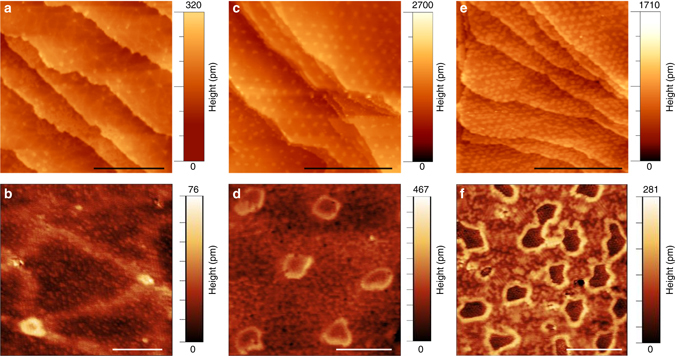
Self-assembled graphene dot arrays in a BCN matrix. **a**, **b** Topographic constant-current STM images of Ir(111) surface covered with a BCN monolayer prepared by co-dosing 31.5 L of borazine (B_3_N_3_H_6_) and 13.5 L of ethylene (C_2_H_4_). BCN domains are surrounded by bright-contrast triangular lines of C-rich regions in a brick-and-mortar pattern. **c**, **d** As C fraction increases (22.5 L of borazine and 22.5 L of ethylene), graphene nanodots of highly uniform size self-assemble and form a 2D superlattice within the host BCN matrix. **e**, **f** As C content increases further (31.5 L of ethylene and 13.5 L of borazine), the dot arrays become denser. The bright rim visible in Fig. 1d, f at the edge of the dots presumably corresponds to the boundary between the dot and the BCN domain. The *scale bars* are 50 nm in **a**, **c**, **e**, 5 nm in **b**, **d**, **f**. (*Imaging parameters*: **a** 0.5 V and 0.8 nA; **b** 0.8 V and 0.2 nA; **c** −1.2 V and 0.9 nA; **d** −0.7 V and 0.3 nA; **e** 0.3 V and 0.2 nA; **f** 0.5 V and 0.4 nA)

Figure 1a shows a scanning tunnelling microscope (STM) image of a BCN monolayer prepared by co-dosing 31.5 langmuir (L) of borazine (B_3_N_3_H_6_) and 13.5 L of ethylene (C_2_H_4_) on a clean Ir(111) surface at 1250 K. The surface is covered by a patchwork consisting of interconnected triangular lines embedded in an apparently featureless background. These lines appear slightly raised relative to the background in the STM image. Sporadically, also small bright-contrast dots are found at the intersections of such lines.

At higher magnifications (Fig. 1b), the background appears as regions of disordered darker and brighter small spots, which covers most of the Ir surface. This structure closely resembles the brick-and-mortar structure reported by Lu et al.^18^, resulting from the exposure of a submonolayer of graphene on a Ru(0001) substrate at 900 K to a low dose of borazine. Notably, the disordered background was identified as a BCN alloy, while the lines were interpreted as segregated graphene ribbons^18^. A similar domain with disordered BCN alloy coexisting with bright-contrast faint lines was previously observed by Sutter et al.^4^, while synthesizing graphene-hBN planar heterostructures on Ru. As we increase the C fraction by co-dosing 22.5 L of borazine and 22.5 L of ethylene, a new arrangement arises within the BCN layer (Fig. 1c, d). A regular array of nanodots self-organises on the Ir surface. In particular, in the close-up image displayed in Fig. 1d, the dots appear to be embedded in the disordered BCN alloy. Interestingly, the arrays maintain the same orientation over different terraces and different areas of the single-crystal substrate. Upon further carbon enrichment of the system (13.5 L of borazine and 31.5 L of ethylene), the distance between adjacent dots decreases, as shown in Fig. 1e, f. Because the nanodots appear when the C concentration in the system is increased and become denser after further C enrichment, we can infer that they are C-rich regions.

**Fig. 2:**
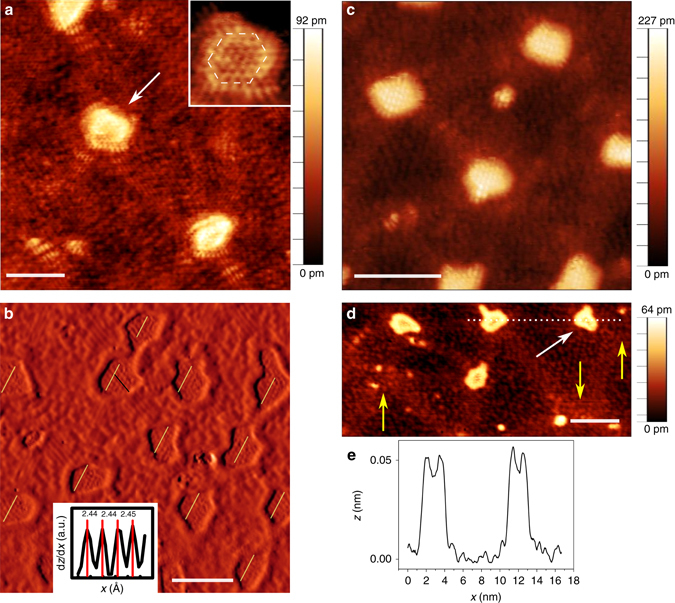
Structural details of nanodots embedded in the BCN matrix. **a** High-resolution STM image highlighting the elongated features present at the edge of the dots and within the mortar lines. The *inset* shows the atomic lattice of the dot marked by the *white arrow*. The dot exhibits hints of hexagonal shape, as highlighted by the *white dashed hexagon* drawn on top (see also Supplementary Note 3). **b** Part of the STM image in Fig. 1f after a first-derivative filter has been applied. Bright areas correspond to positive d*z*/d*x*, and dark to negative d*z*/d*x*, where *x* increases from left to right in the figure. The dots have boundaries largely composed of hexagonal segments; they are preferentially aligned. The *inset* displays the height profile along the *black line* on the dot at the top-centre of **b**. **c**, **d** High-resolution STM images of dots embedded in a BCN matrix highlighting the relationship between the triangular network of C-rich bright-contrast lines and the dots. The *yellow arrows* in **d** pinpoint the locations of missing dots where lines of the triangular network cross each other. The dot highlighted by the *white arrow* is away from the vertex of the triangular network pointed by the *yellow arrow* at right. **e** Line profile taken along the *horizontal dotted line* at the top right corner in **d**, showing two dots and the enhanced apparent height of their edges (that is, the dot rim). The *scale bars* are 3 nm in **a**, **b**, 5 nm in **c**, **d**. Imaging parameters: **a** 0.6 V and 0.3 nA; **c** −0.3 V and 0.7 nA; **d** −0.3 V and 0.5 nA

Figure 2a reveals structural details of the individual nanodots. The *inset* in Fig. 2a displays the dot marked by the *arrow*, which shows a hexagonal shape. In fact, although the dots often have a rather irregular shape, closer examinations suggest that their boundaries are largely composed of hexagonal segments (Supplementary Figs 5–7). By comparing the perimeter length and elongation of the dots with the values for a circle or hexagon (that is, for a compact shape) of the same area, we quantify the degree of irregularity. The ratio of major to minor axis for the best-fit ellipse is 1.5 ± 0.2, while measurements of the perimeter indicate that only half the irregularity is captured by ellipticity (Supplementary Fig. 9). Figure 2b highlights the structural difference between the ordered dot domains and the heterogeneous BCN alloy regions. The honeycomb pattern and uniform density of the atoms in the dots, correlated with their appearance at increased carbon enrichment, suggest that they are graphene dots. This is corroborated by direct measurement of the lattice periodicity within the dots, which is ~0.244 nm (*Inset* of Fig. 2b), in agreement with the value expected for graphene on Ir(111)^19^. More measurements of the lattice periodicity can be found in Supplementary Fig. 3. Moreover, the dots show a preferential alignment as illustrated in Fig. 2b. From Fig. 2c, d it appears that the dot array does not replace the brick-and-mortar structure; rather they coexist. In fact, the dots are generally found at the intersections of the mortar threads. This finding is not very surprising, considering that Lu et al.^18^ interpreted the mortar threads as being graphene nanoribbons. Thus, it is reasonable that the intersections between the threads would be preferential sites for further C segregation. Nevertheless, as illustrated in Fig. 2d, in some cases there is no dot (*yellow arrows*) or the dot is displaced (*white arrow*) from these intersection points. This suggests that the nanodot array is not a simple consequence of the brick-and-mortar pattern. Moreover, the graphene nanodots appear in the STM images with a different contrast than the mortar lines, suggesting a difference between the two structures. Indeed, in Fig. 2c, d, one can notice that, although the mortar lines look brighter than the surrounding BCN areas (that is, the bricks), yet they are neither as bright nor as uniform as the dots. This suggests a non-uniform chemical composition within the mortar structures, in contrast to the nanodots that appear to be pure graphene domains. Furthermore, we also note an increased apparent height at the interface between a graphene dot and the surrounding BCN area, as highlighted by the line profile in Fig. 2e. As we observe this effect only for particular scanning conditions, we ascribe it to electronic effects at the interface between the BCN domain and the pure phase dot.

The non-uniform chemical composition of the mortar lines is also visible in Fig. 2a. Notably, elongated features are clearly noticeable within these lines as well as at some locations at the edge of the dots. These features closely resemble the ones previously associated to B–C bonding states appearing at the interface between graphene and hBN in-plane heterostructures grown on Ir(111), and referred to as lobe-like structures^9^. (B–C bonding motifs are discussed further below.)

**Fig. 3:**
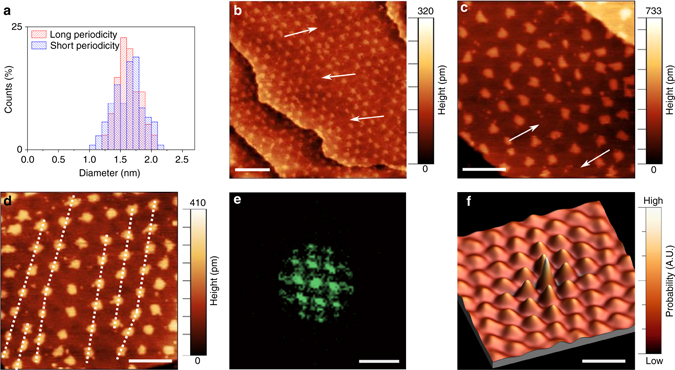
Ordering of the graphene nanodot superlattice. **a** Histogram of the size of the dots. The average dot diameter is 1.60 ± 0.18 nm in the case of the sample with periodicity of 5.9 ± 0.9 nm (long periodicity, *red histogram*), and is 1.63 ± 0.27 nm in the case of the sample with shorter periodicity (3.5 ± 0.7 nm, *blue histogram*). **b**, **c** STM images reporting vacancy-like and **d** dislocation-like defects within the dot superlattice. **e** Fourier transform and **f** 2D autocorrelation image of the STM micrograph in **c**, highlighting the periodicity of the graphene nanodot superlattice. In **f**, *peaks* and *valleys* correspond to distances from a dot at which there is higher or lower probability of finding another dot. The *scale bars* are 20 nm in **b**, 10 nm in **c**, **d**, **f** and 0.4 nm^−1^ in **e**. Imaging parameters: **a** 1.0 V and 0.9 nA; **b**, **c** −1.2 V and 0.2 nA

Figure 3 highlights structural details of the graphene nanodot arrays assembled within the BCN layer. A histogram of the dot size is shown in Fig. 3a for both a long-periodicity dot superlattice and a short-periodicity one (that is, higher C fraction). Examples of these two periodicities are shown in Fig. 1c, e, respectively. The graphene dot size is surprisingly uniform (1.6 nm ± 15%) and insensitive to the dot density; further details of dot size distribution in terms of perimeter and area are reported in Supplementary Fig. 8. Theoretical lines of work suggest that, in graphene dots of this size, quantum confinement opens a band gap somewhat larger than 1 eV, comparable to Si and GaAs^15, 16^.

**Fig. 4:**
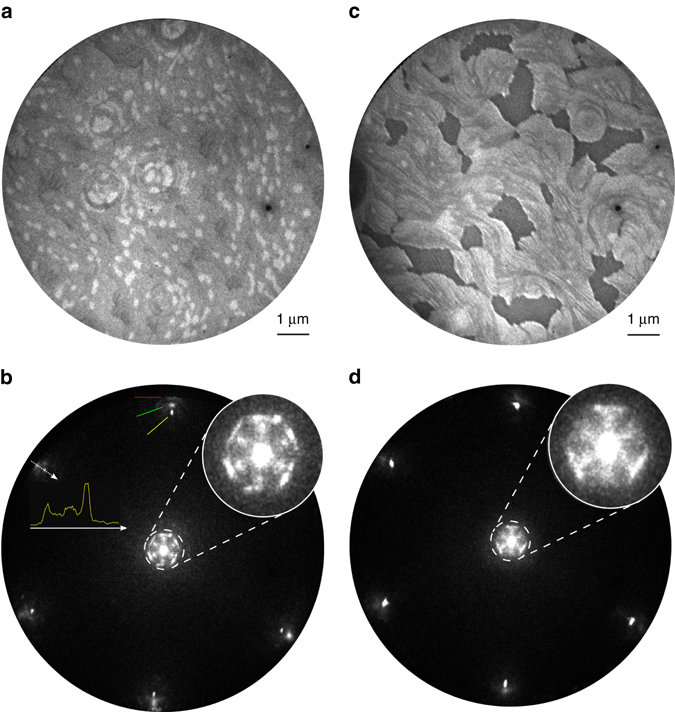
In situ LEEM investigation. **a** LEEM image and **b** µ-LEED pattern of a BCN layer grown by using as precursor gas a mixture of borazine and ethylene. The *small white islands* are graphene seeds grown during the first 120 s of growth, when only ethylene was in the chamber. The *line profile* shown in *yellow* has been taken from the *white arrow* across the (11) diffraction spots of the BCN layer. Following the direction of the *arrow*, the first diffraction spot is due to graphene lattice, the second one to the true BCN domains and the last one to Ir substrate. LEEM image (**c**) and µ-LEED pattern (**d**) of a BCN layer grown by using as a precursor gas a mixture of borazine and ethylene that were pre-mixed prior to being inserted into the growth chamber. The LEEM images were taken with a start voltage of 18.4 V (**a**) and 18.0 V (**c**). The µ-LEED patterns were collected with a start voltage of (**b**) 34 V and (**d**) 47 V

Furthermore, the self-assembled arrays behave like a 2D superlattice, showing characteristic defects such as vacancies and dislocations. For instance, one or more missing dots (that is, single or multiple vacancies) are highlighted in the STM images in Fig. 3b, c by the *white arrows*. Additionally, Fig. 3d shows two edge dislocations, with *white dashed lines* indicating the termination of a row and inward relaxation of the neighbouring ones.

Despite these defects, the graphene nanodot array has surprisingly strong hexagonal ordering, as seen in the sixfold-symmetric pattern in the Fourier Transform (Fig. 3e) of the STM image in Fig. 3d. Autocorrelation is another powerful tool that can be used to characterise the ordering of the nanodot superlattice^20^. Figure 3f displays a 2D autocorrelation function derived from the STM micrograph in Fig. 3d. The regular array of peaks in the autocorrelation confirms the uniformity of the lattice structure across the image. (Further details regarding the autocorrelation analysis are reported in Supplementary Fig. 1).

### In situ low-energy electron microscopy experiments

In situ low-energy electron microscopy (LEEM) experiments were performed in order to gain deeper insight into the growth dynamics of the system. Previous lines of work have already demonstrated the importance of such studies in investigating the growth of graphene–hBN heterostructures on Ru^4, 5^.

Figure 4a shows a LEEM image obtained from a BCN monolayer grown in situ on an Ir(111) crystal. The sample was first exposed to ethylene for 120 s and then to an ethylene–borazine mixture. In the image, bright-contrast islands with diameters of 300–400 nm are embedded in a grey matrix. The bright-contrast islands represent graphene seeds that nucleated during the first 120 s of the growth process, when only ethylene was present in the growth chamber. The grey matrix was formed when both ethylene and borazine were present in the chamber, and it represents the actual BCN layer. During the growth, we observe that BCN starts nucleating preferentially from the edge of pre-existing graphene seeds, that is, according to the so-called lateral epitaxial growth scheme already observed in graphene–hBN heterostructures synthesised by alternating the streams of the respective precursors^5, 21^. The lateral resolution of LEEM is not sufficient to distinguish between different nanoscale phases in the BCN area, but complementary information can be gained by micro-spot low-energy electron diffraction (µ-LEED) analysis, as shown in Fig. 4b. Here one of the six first-order diffraction spots originating from the Ir(111) surface is marked by a *yellow arrow*. Additional weaker diffraction features appear with a lattice constant slightly smaller than the Ir(111): six broad spots with a periodicity in real space of 2.57 Å (marked by the *green arrow*), and six faint, elongated spots at 2.44 Å (marked by the *red arrow*). The 2.44 Å features can be reasonably ascribed to the submicrometre graphene islands observed as bright-contrast areas in the LEEM image in Fig. 4a ^19^. The fact that the diffraction spots exhibit an arc-like morphology implies that the islands have slightly different orientations^22^. We attribute the 2.57 Å spots to the BCN alloy, since they are larger than graphene and h-BN on Ir (2.44 and 2.505 Å, respectively).

**Fig. 5:**
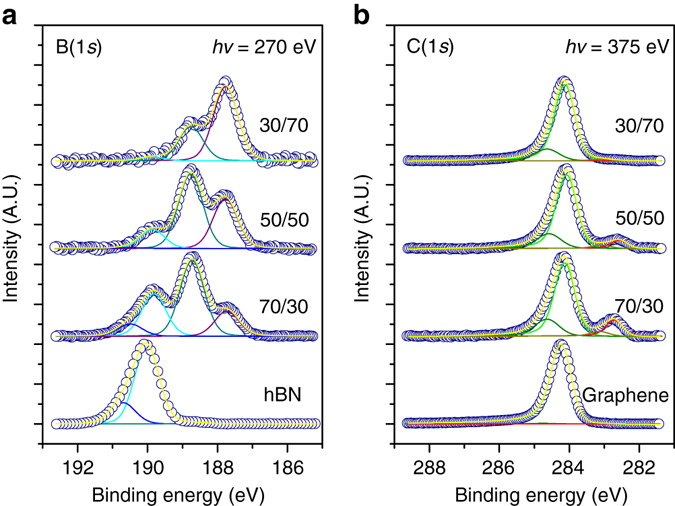
In situ XPS experiments. **a** B(1*s*) core level taken with incident photon energy of 270 eV. **b** C(1*s*) taken with incident photon energy of 375 eV. The *n/m* ratios refer to the partial pressure ratios of borazine, *n*, and ethylene, *m*, used during growth. Data are plotted on separate arbitrary *y*-axes and intensities should not be compared between samples. The experimental data are shown as *empty circles*, while the fit is in *yellow*. The B(1*s*) and C(1*s*) spectra have been fit using four components each. These are labelled B_0_–B_3_ and C_1_–C_4_, respectively. In **a**, the B(1*s*) components have been assigned as follows: B_0_ (*blue curve*) and B_1_ (*cyan curve*) correspond to hBN, with binding energy difference being ascribed to the B_0_ atoms being closer to the Ir(111) substrate. B_2_ (*dark green curve*) represents B atoms binding to at least one C atom in the alloy structure and B_3_ (*purple curve*) are B atoms binding to either B or C, but no to N atoms. In **b**, for the C(1*s*) spectra, the C_1_ (*green curve*) component corresponds to pristine graphene, and C_2_ (*dark green curve*) can be explained by either defects in the graphene or C-N binding motifs. C_3_ (*dark yellow curve*) and C_4_ (*red curve*) correspond to C atoms in the alloy structure binding to one and two B atoms, respectively.The relative intensities for the components of the B(1*s*) and C(1*s*) peaks for each sample are reported in Supplementary Table 2 and Supplementary Table 3, respectively

Interestingly, close to the 00 diffraction spot six bright points with sixfold symmetry appear, followed by six elongated features. The distance of the six points from the 00 diffraction spot indicate a real space periodicity of 3.7 nm. This value is larger than both graphene/Ir and hBN/Ir moiré patterns, which are ~2.5 and 3 nm, respectively. We assign these spots to the periodicity of the graphene nanodot array. Indeed, this value lies within the range measured in the STM experiments for the sample with short periodicity (3.5 ± 0.7 nm), as displayed for instance in Fig. 1e, f. This finding demonstrates that the periodicity of the dot lattice observed with STM is also preserved over macroscopic scales. These six diffraction spots are rotationally aligned with the first-order spots of the Ir surface atoms, suggesting that the formation of the graphene dot array is mediated by interactions with the substrate. The six elongated features appearing close to the above-discussed six spots correspond to a periodicity of ~2.6 nm. This value lies within the range measured for the moiré pattern of graphene/Ir(111)^19^, and most likely originates from the bright-contrast graphene islands observed in the LEEM picture in Fig. 4a. The elongated shape indicates rotational disorder in the moiré pattern, consistent with the rotational disorder already noted in the first-order diffraction spots.

Figure 4c shows a LEEM image of submonolayer BCN grown by exposing the Ir crystal at 1070 K simultaneously to ethylene and borazine, which were pre-mixed before entering the growth chamber. As neither graphene nor hBN seeds are present, we observe that BCN starts nucleating at Ir step edges, and not in the centre of terraces. In the LEEM image in Fig. 4c, bare areas of the Ir substrate appear dark. A few bright-contrast islands of less than 200 nm size can be seen scattered over the surface, and are assigned to graphene areas. We ascribe the dark lines within the BCN domain to height variations in the BCN layer, reflecting steps in the underlying Ir surface. A µ-LEED study of this sample (Fig. 4d) confirms the presence of the inner six spots due to the long-range periodicity of the graphene dots—in this sample the periodicity in real space is 3.4 nm. Three of the six diffraction points are obscured by the threefold outer lines arising from the periodicity of the moiré of the graphene areas.

### In situ XPS experiments

In situ X-ray photoelectron spectroscopy (XPS) using synchrotron radiation was used to give insight into the average chemical composition of the BCN layer. Figure 5 reports XPS measurements of B(1*s*) and C(1*s*) core levels of four samples with varying ratios of partial pressures of borazine to ethylene of 70/30, 50/50, 30/70, plus either pure graphene (used as reference for C(1*s*)) or pure hBN (used as reference for B(1*s*)). The samples were grown with the same partial pressure ratios as the samples displayed in Fig. 1a, b, Fig. 1c, d and Fig. 1e, f, respectively, although a higher total pressure was used during the synthesis of the samples studied with XPS (see Methods). The B(1*s*) spectrum for hBN shows two peaks B_0_ (at 190.60 eV) and B_1_ (at 189.80 eV), which in agreement with previous studies can be both attributed to B–N bonds^23^, with the higher binding energy (BE) peak representing B atoms closer to the Ir substrate^24^. For all other samples the intensity shifts to lower binding energies, fit with peaks B_2_ (at 188.7 eV) and B_3_ (at 187.7 eV) in Fig. 5a. This suggests the prevalence of B–C bonds in the alloyed samples—carbon is less electronegative than N, and similar shifts to lower binding for B–C bonds have been reported elsewhere^25^. On the basis of these previous reports and considering the electronegativity of the three elements at play (namely, C, B and N), we attribute B_2_ to B atoms bound to at least one N atom (either two N and one C, or two C and one N), and B_3_ to B atoms bound to C or B, but not to N (for instance, three C, or two C and one B, and so on). The C1s spectra (Fig. 5b) are dominated, in all cases, by the C_1_ peak. In clean graphene this peak is centred at 284.2 eV, in agreement with values previously reported for *sp* ^2^ carbon in graphene/Ir(111)^26, 27^. The peak shifts to lower binding energies but keeps the same shape in all of the alloyed samples. We attribute this shift to the chemical heterogeneity of the layer and not to a change in the interaction of the graphene with the underlying Ir substrate (Supplementary Fig. 10). However, detailed computational modelling would be required to distinguish between the two. When the gas-growth mixture is weighted with borazine, distinct peaks occur at lower binding energies (C_3_ at 283.2 eV and C_4_ at 282.8 eV). The C_4_ component we ascribe to carbon with two neighbouring B atoms^28^ and the C_3_ component to carbon with only one neighbouring B atom^28^. In all alloyed samples a higher BE shoulder, C_2_, appears at 284.7 eV. This peak may represent *sp* ^3^ defects in graphene^29, 30^ and/or C–N bonding motifs^31, 32^, and it was not possible to distinguish between the two possibilities.

The survival of the intact C_1_ peak and the reduction and disappearance of the components associated with hBN (namely B_0_ and B_1_) in the 50/50 and 30/70 samples rule out the possibility that the nanodots can be hBN regions. In combination with the fact that, in the STM images, the dots appear remarkably different from the disordered alloy in the background, in terms of apparent height and lattice order, this confirms the hypothesis that the nanodots are indeed made of graphene.

The average element distribution across the sample can be estimated from XPS data (Supplementary Table 1) and in all cases carbon was the dominating element. This fits with the observation in LEEM experiments (Fig. 4) that, upon exposure to ethylene, graphene islands nucleate first and grow rapidly on the Ir surface while the borazine partial pressure is being adjusted. Hence, we expect the XPS data for alloyed samples shown in Fig. 5 to represent a mixture of graphene regions and alloyed regions. Using the data in Supplementary Table 1, we can put an upper limit on the fraction of C in the BCN alloy. Assuming that all carbon not in the C_1_ peak originates from C in an alloyed material, and that the most stable BCN systems have B:N ratio of 1^17, 33–36^, we propose a stoichiometry of approximately BC_2.5_N for the alloy matrix when the gas ratio during growth is 50/50. An alloy with such a low percentage of C is expected to be insulating, and thermodynamically stable structures show band gaps in excess of 1 eV in theoretical calculations^17, 32, 36^. A similar calculation for the alloy produced when a borazine-rich gas ratio was used during growth (70/30) gives a stoichiometry of BCN for the alloy. In all alloyed samples, we see some excess of B over N. This probably reflects primarily B atoms dissolved in Ir^23, 37^. In addition, the detailed fitting shown in Fig. 5 suggests excess B at interface between the BCN alloy and the graphene nanodots due to energetic preference for B–C bonds, consistent with previous studies of the interface between graphene and hBN in-plane heterostructures grown on Ir(111)^6, 9^.

## Discussion

The behaviour observed here is strikingly different from that reported in previous studies, where a layer of one pure material (graphene or hBN) was exposed at high temperature to precursors of the other material. Such exposure leads to the formation of an alloy lattice via progressive substitution of atoms, and eventually to complete conversion of graphene to hBN, or vice versa^18, 38, 39^. In particular, Lu et al.^18^ reported a systematic STM investigation of progressive exposure of submonolayer graphene on Ru(0001) to borazine vapour. This exposure initially led to formation of the brick-and-mortar pattern, followed by a patchwork of graphene and hBN domains and eventually complete replacement of graphene by hBN.

It is expected that both the choice of metal and the growth protocol can affect the structure. To clarify the role of the metal, we have repeated the experiment of ref. ^18^ using Ir instead of Ru as the growth substrate. In this case, a mixing of C with B and N is not observed. Instead, on Ir we obtain interconnected pure phase graphene–hBN heterostructures with sharp interfaces (Supplementary Fig. 2), consistent with similar lines of work of sequential gas exposure on Ir^6, 9^. The difference most likely results from differences in the catalytic properties of Ir and Ru. Indeed, Sutter et al.^5^ showed that the same procedure for growing 2D heterostructures applied to different substrates may lead to entirely different results. Nevertheless, it is worth noting that graphene dot arrays might also occur for the substitution reaction on Ru(0001). Although it was not reported in ref. ^18^, their images of the intermediate stages of transformation show features that might be graphene dots. On the other hand, the periodicity of those features could also be attributed to the moiré of graphene/Ru(0001). If further investigations should prove that dots can form on Ru, it would support the generality of the behaviour that we observe.

Here, in contrast to previous work, which used sequential dosing, we simultaneously dose ethylene and borazine on an Ir(111) surface. As discussed above, only with such co-dosing do we observe graphene nanodots. While the mechanism is not clear, it seems reasonable that co-dosing could facilitate the self-organisation into discrete graphene dots within the BCN host phase. Sequential dosing requires nucleating graphene regions in pre-existing hBN, and, if the nucleation barrier is large, that could overwhelm any thermodynamic driving forces for self-organisation.

What is most remarkable is the degree of order observed, both in the dot-size uniformity and the spatial distribution. This surprising behaviour requires some discussion. At first glance, the dot formation and ordering seem strikingly similar to that predicted to arise driven by elastic relaxation in 2D domains of different stress^13, 14^ (or electrostatic interactions between domains of different work functions^13, 14^). In that case, if the fractional area of each phase is fixed, the equilibrium configuration can be a hexagonal lattice of compact regions (dots) of the minority phase. The size of the dots is then determined by a competition between interfacial line energy (favouring large dots) and stress-relaxation energy (favouring small dots). However, we note that the graphene dots here have roughly the same size as the features in the moiré pattern of graphene on Ir(111). This suggests that the substrate interaction might play a dominant role in determining the size of the dots. Otherwise, the dot size is expected to vary exponentially with boundary energy^13, 14^; therefore, it would be an improbable coincidence if that physics led to dots of similar size to the graphene moiré features.

To gain qualitative insight into the dot formation, we consider what dot size will minimise the energy, for a fixed area of graphene embedded in BCN. For now we neglect elastic energy; the effect of this and other approximations is discussed below. The energy of a circular dot of radius *R* is then

1 $${E_{{\rm{dot}}}} = 2\pi R\lambda + {\int} {U\left( {\bf{r}} \right){\rm{d}}{\bf{r}}} $$

where *λ* is the energy per length of the graphene–BCN boundary, *U* is the graphene–substrate interaction, **r** is a 2D vector and the integral is taken over the dot. The interaction *U* varies laterally because of the varying alignment between graphene and substrate atoms, as reflected in the moiré pattern. (The BCN shows no moiré pattern, so we neglect the corresponding variation in interaction energy for BCN.) The simplest possible form for the interaction is

2 $$U\left( {\bf{r}} \right) = {U_0} - \alpha \mathop {\sum}\limits_{\bf{g}} {\left[ {\cos \left( {{\bf{g}} \cdot {\bf{r}}} \right)} \right]} $$

Here *U*_0_ is the average interaction, that is the interaction energy per unit area for a large graphene domain; *α* is the amplitude of the variation; and the three **g** vectors of magnitude 2*π*/*L*_m_, 120° apart, describe the hexagonal moiré pattern, which has period *L*_m_ = 2.5 nm for graphene on Ir. This interaction energy is minimised at a hexagonal lattice of points, and has approximate rotational symmetry up to distances of ~0.4 *L*_m_ (i.e., 1 nm) from each minimum.

**Fig. 6:**
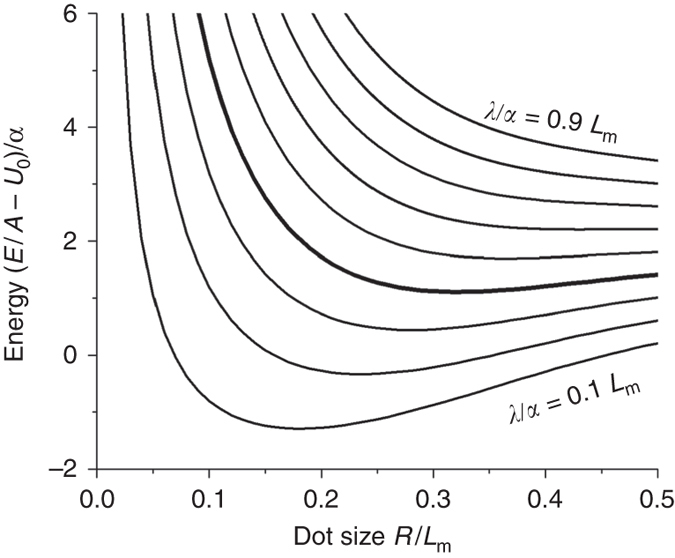
Energy vs. dot size, in units of *α*, from Eqs. (1 and 2). The energy is calculated per unit area of graphene, while the dot size is in units of moiré periodicity (*L*_m_)*. Curves* correspond to different values of *λ*/*α*, ranging from 0.1*L*_m_ (*bottom*) to 0.9*L*_m_ (*top*). The heavier curve (*λ*/*α*  = 0.4*L*_m_, fourth from *bottom*) gives a dot radius close to the experimentally observed size. For values of *λ*/*α* greater than around 0.6*L*_m_ (*sixth curve* from bottom), there is no minimum at any *R*, suggesting that dots will not form

Assuming a fixed total area of graphene, we want to minimise the dot energy per area, *E*_dot_/(πR^2^). It is convenient to focus on the dot energy/area, relative to a large graphene domain, that is [*E*_dot_/(πR^2^)−*U*_0_], which is independent of *U*_0_. This is plotted in Fig. 6, in units of *α*, for different values of the boundary energy *λ* (or rather, of the ratio *λ*/*α*). We see that there are three distinct regimes. For sufficient small boundary energy, there is an energy minimum at dot sizes corresponding to a small fraction of the moiré period (small *R*/*L*_m_), with energy lower than a uniform graphene sheet. Thus, dots are the stable phase in this case. For larger boundary energy, there is still a minimum but at positive energy values; therefore, dots are metastable. They could easily form if the carbon mobility is limited. For still larger values of boundary energy, there is no minimum at any finite *R*, and dot formation cannot occur within our model. (It is worth noting that Eqs. (1 and 2) could apply equally well to graphene dots on Ir without a BCN host matrix. However, in that case the free edge would have dangling bonds, giving such a high value of edge energy *λ* that dots would be unstable.)

The experimentally observed dot size, *R* = 0.8 nm = 0.32*L*_m_, corresponds to the range where dots are robustly metastable; therefore, it is worth discussing this regime in more detail. Such dots are stable against coarsening—if they form with a range of diameters, any diffusion will actually drive the distribution to become more uniform. This is true as long as the distribution is confined to a limited range of *R*, as in our experiments. However, for a sufficiently large graphene domain, the energy/area drops towards zero. Therefore, within diffusion range of such large domains, the dots will shrink and disappear as material diffuses to the larger domains. Thus, metastable dot arrays are expected to form under conditions where, kinetically, there is no opportunity for the formation of very large domains nearby.

Of course, this simple model neglects many factors that must also play a role. In particular, the organisation of the dots into a hexagonal array is consistent with the long-range repulsion expected to arise from elastic and/or electrostatic interactions^14^. These interactions also affect the dot self-energy. However, if they are not too strong, they can be thought of as mainly acting to renormalise *λ*, with only a weak dependence of the effective *λ* value on *R* ^14^. The fact that the dot array is always oriented along the substrate lattice directions, as shown in the LEED images in Fig. 4b, d, presumably reflects an anisotropy imposed by the atomic structure of the substrate. The BCN must also interact with the substrate, and, while the alloy randomness may wash out any alignment-energy contribution, as it washes out any BCN/Ir moiré pattern, the lateral variations may not be negligible. Nevertheless, it should at least be smaller than that for graphene (which shows a strong moiré pattern on Ir); therefore, the *α* term may be interpreted as a net benefit from making the graphene optimally aligned. Also, high-resolution STM images suggest that the actual dot shape is not circular, but rather irregular with considerable hexagonal character. This does not change the qualitative picture above, but the coefficient of the edge energy will depend on the actual shape.

Theory suggests that our system corresponds to semiconducting quantum dots^15, 16^ epitaxially embedded in a larger-bandgap BCN matrix^17^. Nevertheless, the strong coupling with the underlying Ir substrate (Supplementary Fig. 10) modifies the intrinsic electronic properties of the BCN layer, and possible quantum confinement effects cannot be observed. In order to unambiguously determine the electronic properties of this novel material and exploit it for applications, it will be necessary to transfer it from the growth substrate. Although challenging, successful transfer has already been achieved for graphene–hBN heterostructures^5^ and for BCN layers^32^; therefore, one can assume that it may be achieved for this system as well.

In conclusion, STM, LEEM and XPS have been used to show the self-assembly and self-organisation of ordered 0D graphene dots epitaxially integrated within a 2D BCN monolayer. These nanodots exhibit a strikingly uniform size, a consistent lattice alignment and strong hexagonal ordering over large areas, with periodicity that can be tuned via the growth conditions. The ordering can be understood as resulting from the presence of long-range repulsive interactions among the dots, while the uniform dot size is explained by a model taking into account the dot-boundary energy and a moiré-modulated interaction with the Ir substrate. This substrate interaction presumably also drives the lattice alignment among the graphene nanodots. The present work paves the way for an extreme form of material design, providing a radically new material that is expected to have novel optoelectronic properties and a variety of potential device applications, with the additional flexibility of tuning the superstructure through control of the growth conditions. Because the behaviour in our system results from a simple competition of edge energy and moiré-modulated substrate interaction, it might also occur for heterostructures of other 2D materials on appropriate substrates.

## Methods

### Sample preparation

Data were collected in different ultrahigh vacuum chambers but following the same recipe for sample preparation in each. An Ir(111) crystal was cleaned by several sputter/anneal cycles using 2.5 keV Ne^+^ ions, followed by annealing in an oxygen atmosphere (3 × 10^−7 ^mbar) to remove carbon contaminants. The clean Ir surface was exposed to a gas flux of ethylene and borazine, following standard background dosing technique, for 15 min at 1250 K. In all cases, aside from the LEEM experiments detailed below, the gases were not premixed prior to their introduction into the chamber and were therefore introduced one at a time. The ethylene/borazine ratio was controlled by adjusting the partial pressures of the individual gases to give a higher or lower ratio as required. We do not quote the absolute pressures used as variations between the chamber geometries and pressure gauge calibrations do not allow for precise determinations but typically the samples were exposed to a total background gas pressure of between 0.5 and 1 × 10^−7^ mbar.

### STM characterisation

STM experiments were performed on a Createc low-temperature STM at 77 K with a base pressure below 10^−10^ mbar. Pt–Ir tips were used. STM images were processed using WSxM^40^ and Gwyddion^41^ software. The diameter of the graphene dots was estimated from the STM images, by assuming that the bright rim (see Fig. 1d, f) at the edge of the dots corresponds to the border between the dot and the BCN domain.

### In situ LEEM experiments

Bright-field LEEM mode in an Elmitec aberration-corrected LEEM was used to observe the growth of the BCN layer in situ. A C-type thermocouple was used for reading sample temperature. The growth temperature was 1070 K. Selected-area electron diffraction was acquired with an aperture of 1.5 µm.

### In situ XPS experiments

The XPS experiments were performed at the *Matline* beamline of the ASTRID II synchrotron radiation facility (Aarhus, Denmark). The beamline is equipped with a SX-700 monochromator, allowing for light in the energy range of 20–700 eV. The end station consists of a single chamber, equipped with a *Scienta* electron energy analyser, and runs at a base pressure of <10^−9^ mbar during data acquisition. An Ir crystal was cleaned and the absence of any C, B, O and N contaminants was checked by XPS. Samples were prepared as described above, although during the growth process a higher total background gas pressure was used (0.5–1 × 10^−6^ mbar). The C(1*s*), and B(1*s*) core levels were collected in normal emission geometry (incidence/emission angles of 0°/45°) with photon energies of 375 and 270 eV, respectively. The BE was calibrated by measuring the Fermi edge for each photon energy. The C(1*s*) spectra for each sample were fit with 4 Doniach-Šunjić components convoluted with Gaussians. The Lorentzian full width at half maxima (FWHM) were fixed at 130 meV for each component and the Gaussian FWHMs were allowed to change between components. To account for doping of the graphene film, the BE position of the C_1_ component was allowed to change, while the relative positions of the C_2_, C_3_ and C_4_ components were fixed at +0.503, −1.413 and −0.992 eV, respectively. The B1s spectra were fit with 4 Voigt components using the same Lorentzian and Gaussian FWHM, 100 and 770 meV, respectively, for each component. Analogous to the C(1s) spectra, also in the case of the B1s core level, the B_0_ component was allowed to change in BE while the relative positions of the B_1_, B_2_ and B_3_ components were fixed at +0.680, −1.093 and −2.087 eV, respectively.

### Data availability

The data sets generated during and/or analysed during the current study are available in the Zenodo repository (doi: 10.5281/zenodo.569453).

## Electronic supplementary material

Supplementary Information
