# Erratum: Self-assembly of ordered graphene nanodot arrays

**DOI:** 10.1038/s41467-017-00725-y

**Published:** 2017-10-24

**Authors:** Luca Camilli, Jakob H. Jørgensen, Jerry Tersoff, Adam C. Stoot, Richard Balog, Andrew Cassidy, Jerzy T. Sadowski, Peter Bøggild, Liv Hornekær

**Affiliations:** 1 Center for Nanostructured Graphene, DTU Nanotech, Technical University of Denmark, Kongens Lyngby, DK-2800 Denmark; 2 Department of Physics and Astronomy and Interdisciplinary Nanoscience Center iNANO, Aarhus University, Aarhus C, 8000 Denmark; 3 IBM T.J. Watson Research Center, Yorktown Heights, New York, NY 10598 USA; 4 Center for Functional Nanomaterials, Brookhaven National Lab, Upton, NY 11973 USA

*Nature Communications* **8**:47 doi:10.1038/s41467-017-00042-4; Article published online 29 Jun 2017

An incorrect version of the Supplementary Information was inadvertently published with this article where the wrong file was included. The HTML has been updated to include the correct version of the Supplementary Information.

