# Supplementary material for: Self-assembly of ordered graphene nanodot arrays
Source: Nat Commun. 2017 Jun 29;8:47. doi: 10.1038/s41467-017-00042-4 (PMC5491516; doi:10.1038/s41467-017-00042-4)
Supplement: Supplementary file 1 — Supplementary Information [file 41467_2017_42_MOESM1_ESM.pdf]

Description of Supplementary Files

File Name: Supplementary Information
Description: Supplementary Notes, Supplementary Figures, Supplementary Tables and Supplementary References.

Supplementary Note 1. Autocorrelation analysis of graphene nanodot superlattice

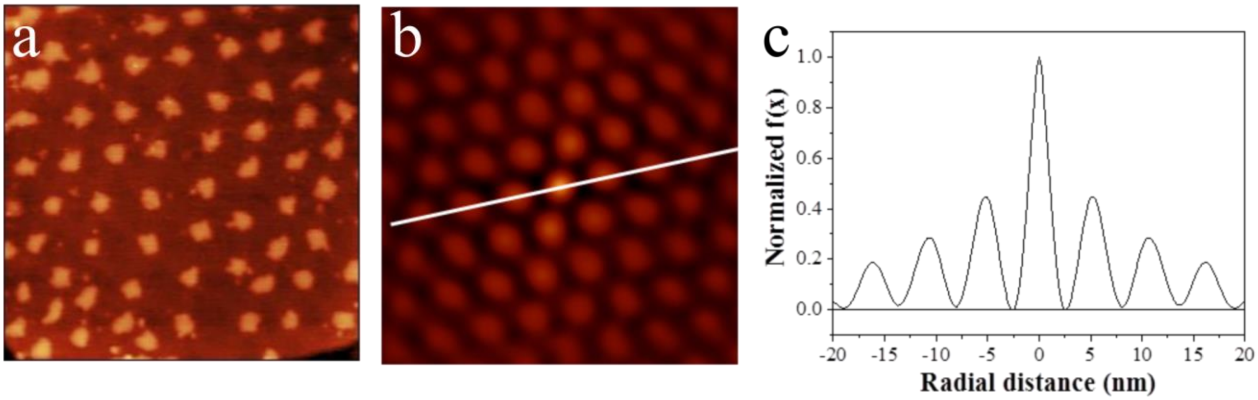

Supplementary Figure 1. Autocorrelation analysis.(a) 41x41nm scanning tunneling microscopy (STM) image of graphene nanodots self-assembled in a BCN matrix; (b) 2D autocorrelation image of the STM picture in (a); (c) line profile along the white line in the 2D autocorrelation image, showing the normalized radial distribution function (f(x)). (Imaging parameters for STM image: -1.2 V and 0.2 nA)

Supplementary Note 2. Growth and STM characterization of graphene-hexagonal boron nitride (hBN) in-plane heterostructures synthesized by alternating the streams of the relative precursors

We have investigated the possibility of creating BCN alloy structures by alternating the streams of the gas precursors (i.e., ethylene and borazine), as a comparison to the study by Lu et al. performed using Ru(0001) as the growth substrate[1].

Exposing a complete monolayer (ML) of graphene on Ir(111) to 5 langmuir (L) of borazine vapour at 1000 K led to formation of neither BCN alloy nor in-plane graphene-hBN heterostructures. Indeed, no visible change to the graphene layer was observed after STM investigation. This is most probably due to the fact that the complete monolayer of graphene protected the Ir substrate which hindered the decomposition of borazine.

Next, we exposed 0.8ML of graphene to 5 L of borazine vapor at 1000 K. STM analysis showed areas of bare Ir and some with small hBN islands in addition to the graphene regions. No BCN alloy was observed. Then, we increased the borazine dose by 12L at a growth temperature of 1200K. The formation of in-plane heterostructures of pure graphene and hBN was achieved (Supplementary Figure 2). Furthermore we noticed a small decrease in graphene coverage probably due to etching from atomic hydrogen formed during the decomposition of borazine molecules. In conclusion, we could not see any alloying between C, B and N on Ir by using a growth method consisting of alternating the stream of the gas precursors.

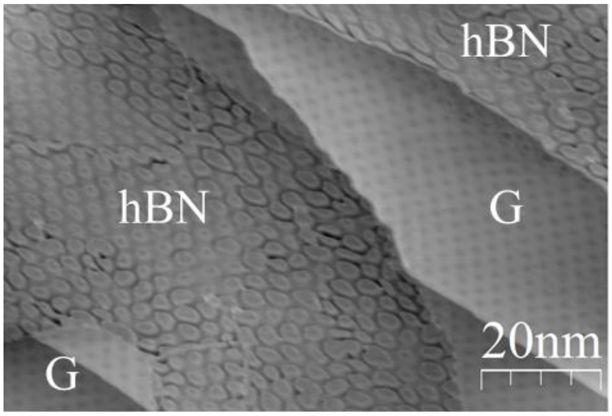

Supplementary Figure 2. Graphene and hBN planar heterostructures on Ir(111). STM image of graphene-hBN heterostructures formed after exposing 0.8ML graphene/Ir to 17L of borazine vapor. Sharp interfaces between hBN and graphene islands are shown, while no BCN alloying is observed. (Imaging parameters: 0.45 V and 0.1nA).

Supplementary Note 3. Additional structural characterization of graphene nanodots and BCN alloy

Atomically resolved STM pictures have been used to provide insight into the structure of graphene nanodots and the surrounding BCN alloy. The lattice spacing, orientation, size and shape of the graphene nanodots can be characterized in great detail. Below, STM images of the nanodot phase allow, for instance, determination of lattice constants in agreement with those expected for graphene. Structural characterization of the BCN-alloyed regions, however, is more difficult because of the intrinsic elemental inhomogeneity. In topographic images, the disordered bright and dark spots within the BCN-alloyed regions dominate the contrast. Only after filtering the STM images, it is possible to resolve locally the honeycomb structure of the BCN alloy. Filtering techniques applied to selected images have also been used to demonstrate the structural properties of the two different types of region; the ordered graphene nanodots and the heterogeneous BCN alloy. The STM data have been processed using WSxM[2](for first derivative and laplacian filters) and Gwyddion[3](further analysis).

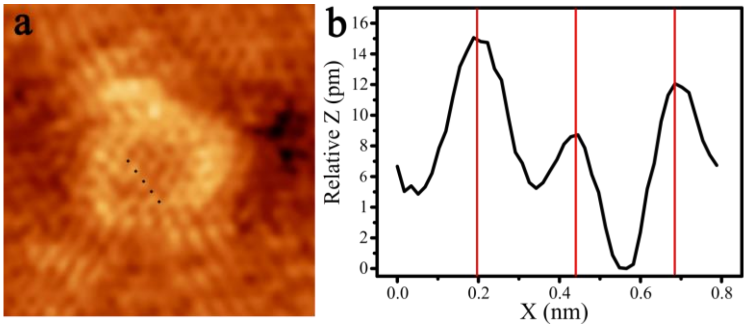

Supplementary Figure 3. Lattice parameter of a graphene nanodot. (a) Atomic resolution STM image of the graphene dot displayed in the inset of Fig. 2a in the main text. (b) The line profile taken across the black dotted line in the STM image in (a). The average value is 0.243 nm, close to the 0.244 nm expected for graphene on Ir(111)[4].

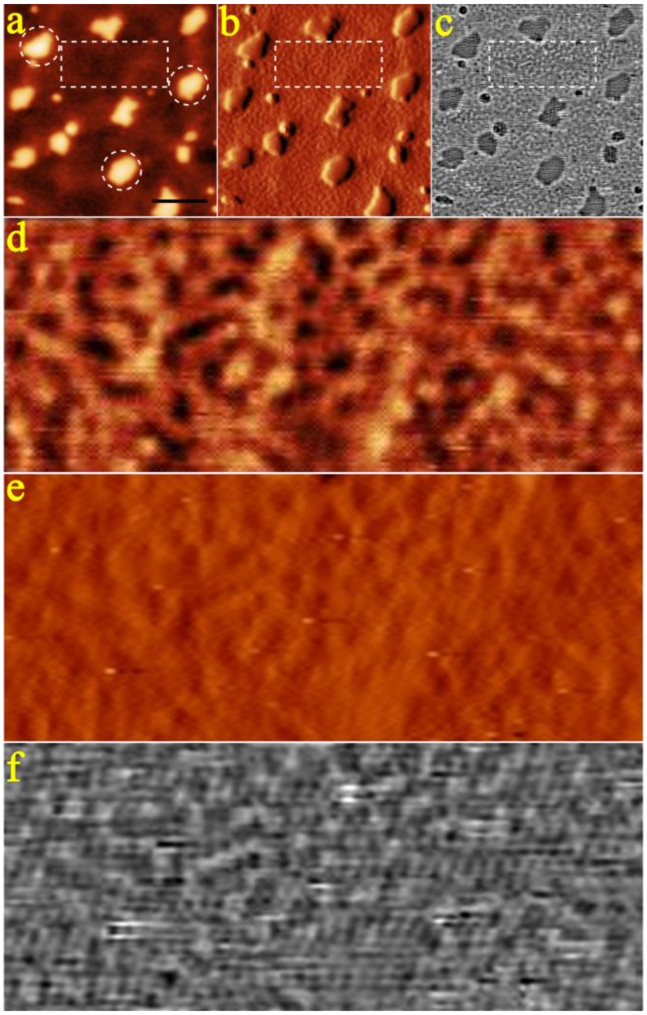

Supplementary Figure 4. Structural details of the BCN alloy. (a) Unfiltered STM topographic image, (b) same image after first derivative, and (c) after laplacian filters. The scale bar is 5nm. (d), (e) and (f) are the zoom-in of the area highlighted by the dashed rectangle in (a), (b) and (c) respectively. Only after laplacian filtering (f) it is possible to observe areas showing locally the honeycomb lattice of the BCN alloy (middle right side of the micrograph).

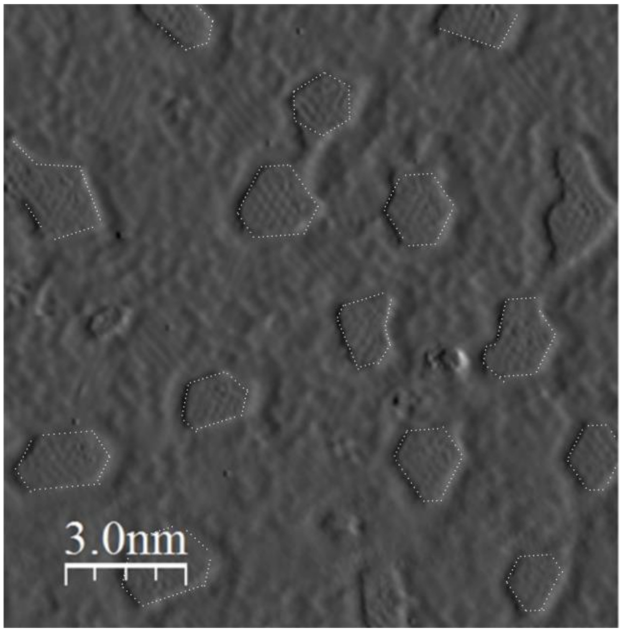

Supplementary Figure 5. Shape of nanodots. First-derivative of the image in Fig 1f in the main text. Although some of them exhibit a rather irregular shape, the majority of the dots are hexagonal, but often with unequal edge lengths and other deviations. Dotted lines have been drawn around the edge of some of the dots to highlight their shape.

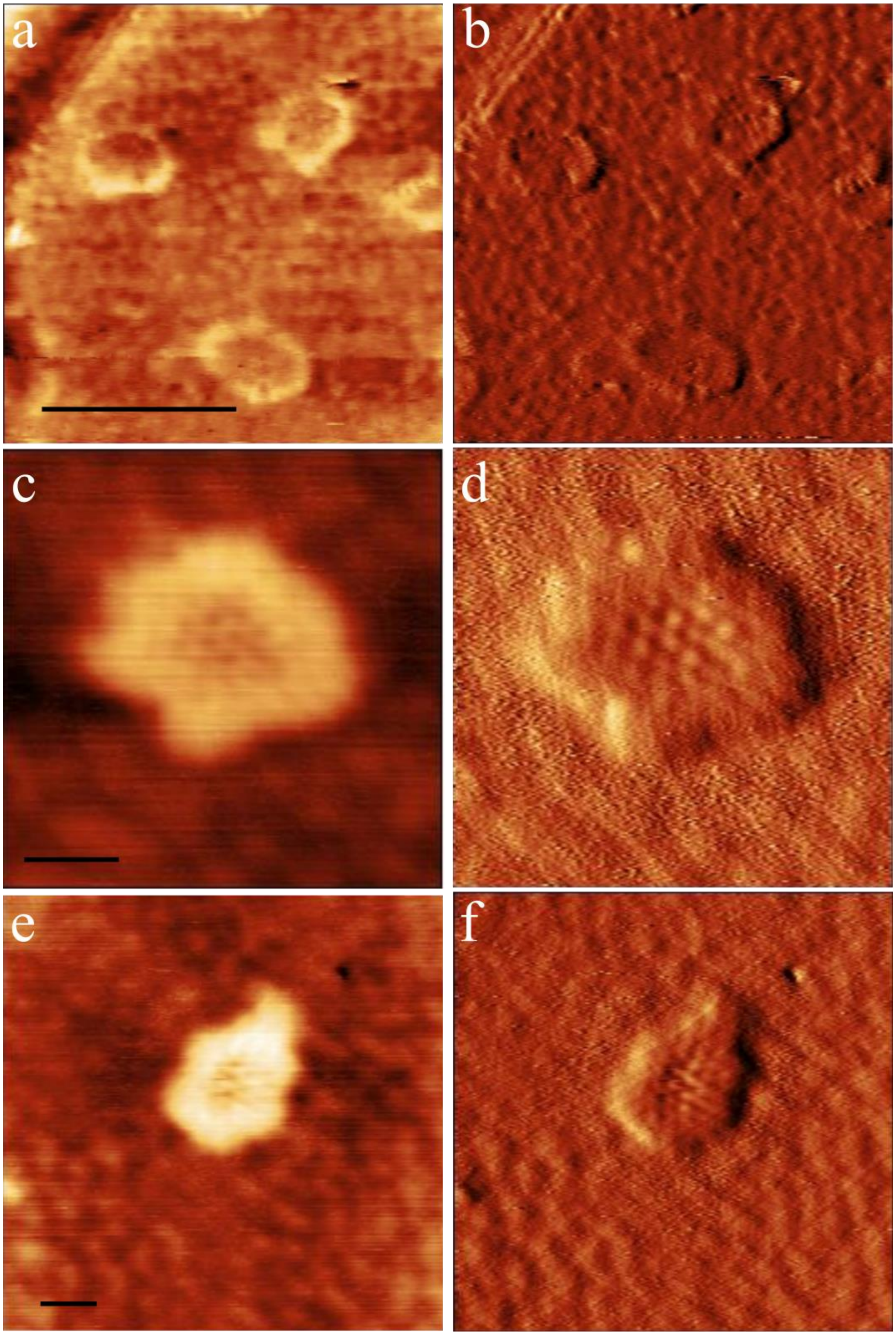

Supplementary Figure 6. Shape of nanodots. Collection of unfiltered (a, c, e) and filtered (b, d, f) STM images. The dots in these images all exhibit a shape with hexagonal characteristics. Scale bars: 4 nm (a, b); 1 nm (c-f).

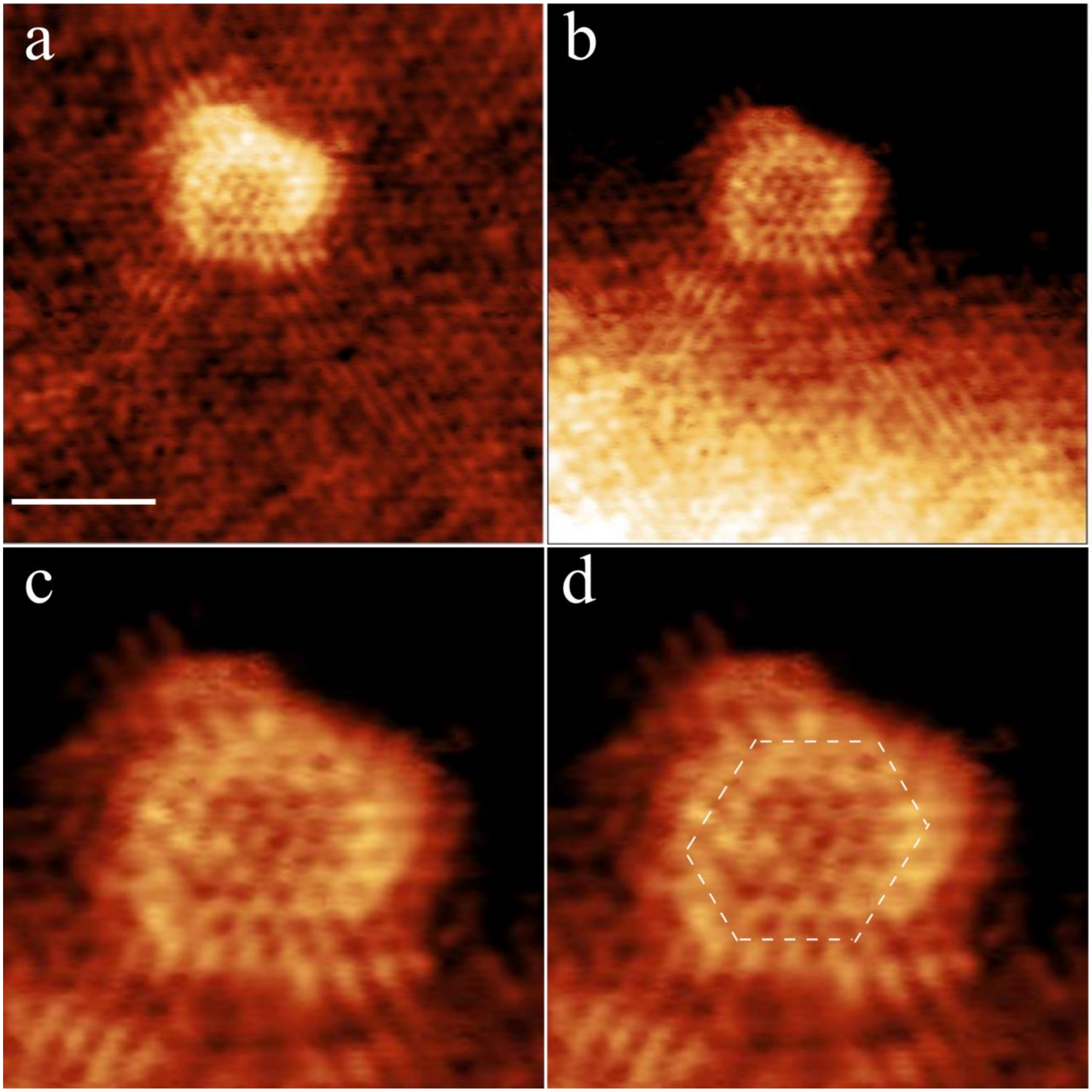

Supplementary Figure 7. Shape of nanodots. (a) Detail of the STM image displayed in Fig. 2a of the main text, showing the dot which is illustrated in the inset of Fig. 2a in the main text. (b) Same image, after the background has been removed in such a way to make even the contrast within the dot. (c) Zoom-in view of the dot, which shows a hexagonal shape, as highlighted by the dashed lines drawn on top in (d). Scale bar: 2 nm.

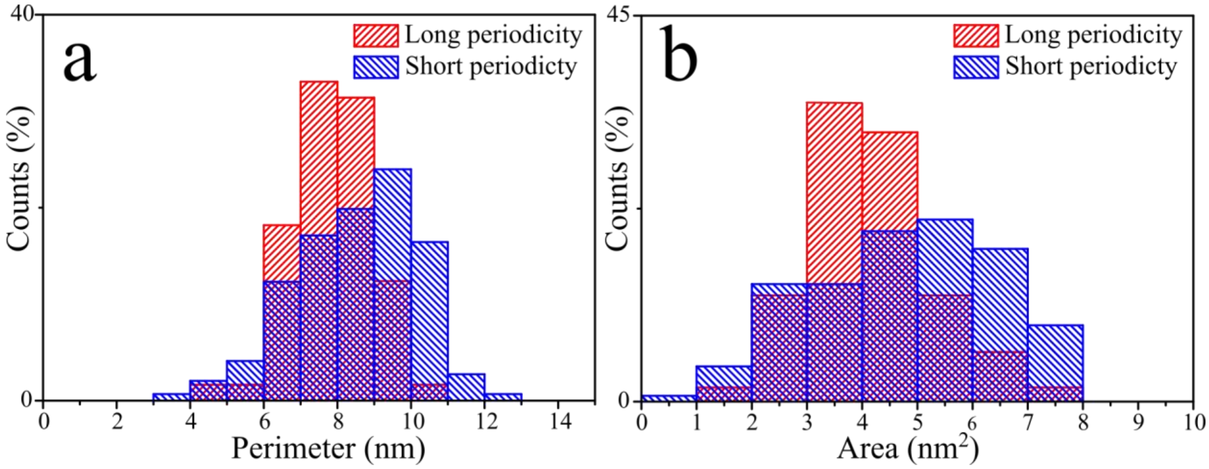

Supplementary Figure 8. Histogram of perimeter and area of the graphene dots in the samples with long and short periodicity. The perimeter and area have been estimated by using *ImageJ* software. The average values (normal distribution) of the perimeter are 7.8 nm ± 1.1 nm for the sample with long periodicity and 8.5 nm ± 1.6 nm for the short periodicity one. The average values (normal distribution) for the area are 4.1 nm ± 1.1 nm for the long periodicity sample and 4.7 nm ± 1.6 nm for the short periodicity one.

In order to provide quantitative information about the shape of the graphene dots, we have quantified how far they deviate from a compact shape. We have calculated the area and perimeter of several dots in different high resolution STM images (due to the small size of the dots, only high resolution images allow for a fine measurement of these features), then for each area, we have calculated what is the perimeter of a (ideal) hexagon with that area. The ratio between the actual perimeter and the ideal perimeter gives a measure of how irregular the dot is, with 1 being a hexagon, and >>1 being extremely irregular. As not all the dots show clearly a hexagonal shape, we have then also used a similar approach to compare the perimeter of the best-fit ellipse and an ideal circle. This again informs about how far the dot shape deviates from a compact shape. These data are collected in the histograms in Supplementary Figure 9, along with a histogram for the ratio of major to minor axis for the best-fit ellipse.

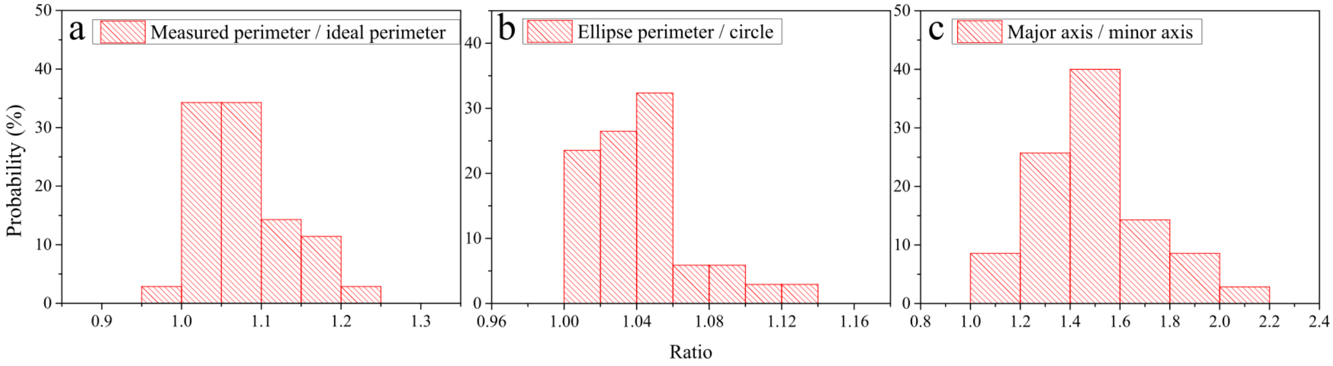

Supplementary Figure 9. Quantification of dot shape. (a) histogram of the ratio of measured graphene dot perimeter to (ideal) equivalent hexagon perimeter (average value: 1.08±0.06). (b) : histogram of the ratio of best-fit ellipse perimeter to circumference of equivalent circle (average value: 1.04±0.03). (c) Histogram of the ratio of major to minor axis of best-fit ellipse (average value: 1.5±0.2).

From the data above we can conclude that the elongation (elliptical vs compact shape) captures much of the deviation from ideal circles or hexagons, but the islands are still significantly more irregular than the best-fit ellipse, as evidenced by the actual to ideal perimeter ratio (Supplementary Figure 9a, average being 1.08±0.06) being higher than the perimeter ratio for the best-fit ellipse to same-area circle (Supplementary Figure 9b, average being 1.04±0.03).

Supplementary Note 4. X-ray photoemission spectroscopy

In order to estimate the amount of B, C and N on the surface for each sample, we have collected the B(1*s*), C(1*s*), Ir(4*d*) and N1s photoelectrons emitted when samples were irradiated with the same incident photon energy (610 eV). By growing full ML coverage of graphene (hBN) on Ir and setting the resulting C/Ir ratio equal to 1 (B/Ir = 0.5 and N/Ir = 0.5) we can work out the corresponding abundance of these elements in alloyed samples. The relative amount of the individual elements is reported in the Supplementary Table 1. As referred to in the main text LEEM experiments show that upon exposure to ethylene, graphene flakes nucleate and grow first on the Ir surface while the borazine partial pressure is still being adjusted. Hence we expect the XPS data for alloyed samples to represent a mixture of graphene flakes and alloyed regions. The abundance of C can then be partially allocated to regions with fast growing graphene flakes in all samples. In all alloyed samples there is an excess of B over N. This finding can be explained by (i) the energetic preference for B-C bonds at interfaces between BCN and graphene regions (in analogy to what is observed at the interface between hBN and graphene islands grown on Ir(111)[5,6]), and (ii) the presence of small quantities of B dissolved in the Ir crystal[7,8].

Ultimately, we have characterized the interaction between the BCN layer and the Ir substrate, by looking at the Ir(4$f_{7/2}$) core level (Supplementary Figure 10).

	**B/Ir**	**C/Ir**	**N/Ir**
**70/30**	0.21	0.31	0.11
**50/50**	0.17	0.56	0.06
**30/70**	0.03	0.78	0.01

Supplementary Table 1. Average element distribution. The average element distribution for the samples grown with borazine/ethylene ratios of 70/30, 50/50 and 30/70 is shown.

	B_0	B_1	B_2	B_3
**hBN**	21.28	77.44	1.28	0.00
**70/30**	7.87	27.27	48.79	16.07
**50/50**	0.65	13.12	51.71	34.52
**30/70**	0.00	3.38	30.30	66.31

Supplementary Table 2. Relative intensity of the individual components of the B(1*s*) peak. The relative intensity of each component of the B(1*s*) peak for the samples grown with borazine/ethylene ratios of 100/0 (that is, hBN), 70/30, 50/50 and 30/70 is shown. For each sample, the intensities of the individual components are normalized to 100.

	C_1	C_2	C_3	C_4
**Graphene**	98.72	0.78	0.50	0.00
**70/30**	65.91	15.30	4.28	14.50
**50/50**	73.58	14.73	3.42	8.27
**30/70**	84.83	13.04	1.75	0.38

Supplementary Table 3. Relative intensity of the individual components of the C(1*s*) peak. The relative intensity of each component of the C(1*s*) peak for the samples grown with borazine/ethylene ratios of 0/100 (that is, graphene), 70/30, 50/50 and 30/70 is shown. For each sample, the intensities of the individual components are normalized to 100.

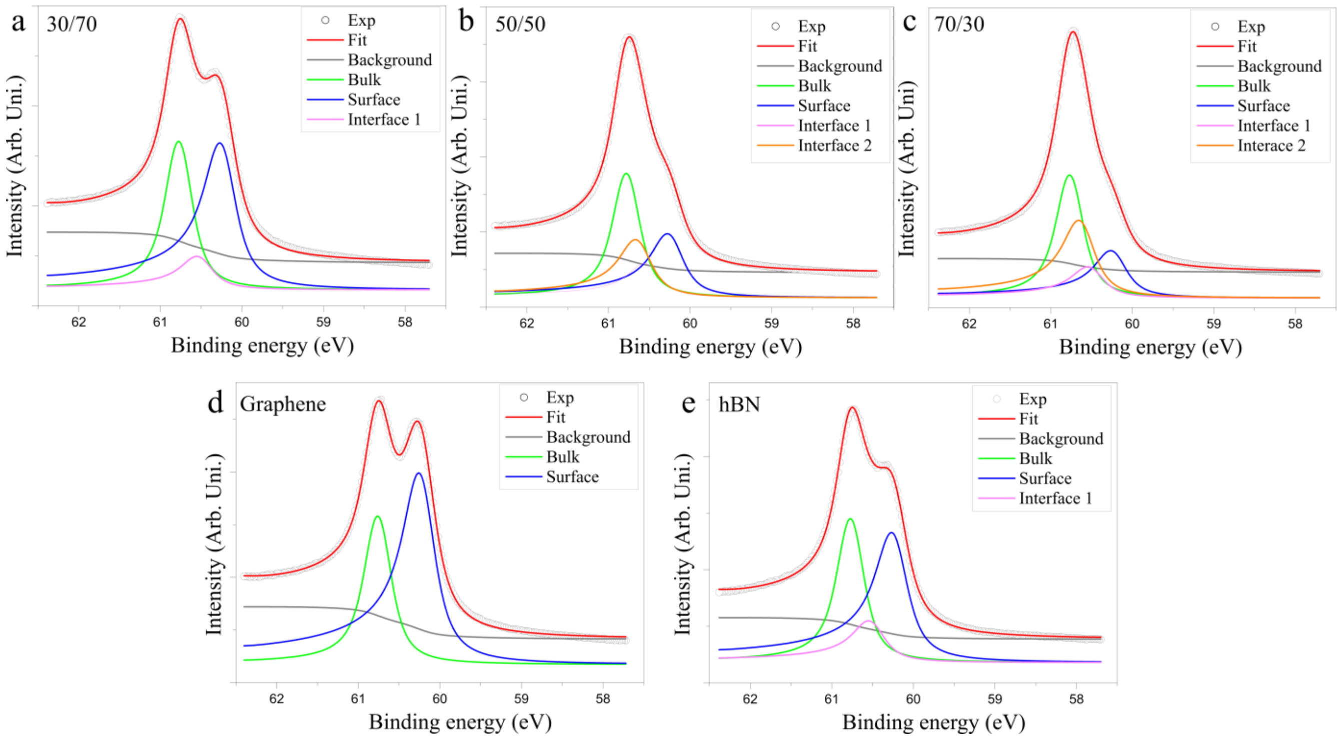

Supplementary Figure 10. Ir $4f_{7/2}$ of the samples grown with borazine/ethylene gas ratios of 30/70 (a), 50/50 (b), 70/30 (c), 0/100 (graphene, d) and 100/0 (hBN, e) obtained with an incident photon energy of 120 eV. The interaction between graphene and the Ir

substrate is weak, and the Ir $4f_{7/2}$ can be fit using two, namely bulk and surface, components. In the case of hBN, the interaction with the substrate is stronger, so that the surface component splits into the clean surface component as used to fit the graphene data and a new surface component, Interface 1. The Interface 1 peak represents Ir surface atoms bound strongly to B and N atoms in the overlying hBN layer[7,9]. The 30/70 sample can be fit using the three components (that is, the bulk peak, the clean surface component and the interface 1 component). The 50/50 and 70/30 samples require introduction of a second interface component, Interface 2, to fit accurately the data. The second Interface component again represents surface Ir atoms bound to the overlying layer.

Supplementary References

1. Lu, J. *et al.* Order-disorder transition in a two-dimensional boron-carbon-nitride alloy. *Nat Commun* **4**, 2681 (2013).
2. Horcas, I. *et al.* WSXM: A software for scanning probe microscopy and a tool for nanotechnology. *Rev. Sci. Instrum.* **78**, 013705 (2007).
3. Nečas, D. & Klapetek, P. Gwyddion: an open-source software for SPM data analysis. *Open Physics* **10**, 181-188 (2012).
4. Alpha, T. N. D., Johann, C., Tim, N. P., Carsten, B. & Thomas, M. Structure of epitaxial graphene on Ir(111). *New J. Phys.* **10**, 043033 (2008).
5. Drost, R. *et al.* Electronic states at the graphene–hexagonal boron nitride zigzag interface. *Nano Lett.* **14**, 5128-5132 (2014).
6. Liu, M. *et al.* Quasi-freestanding monolayer heterostructure of graphene and hexagonal boron nitride on Ir(111) with a zigzag boundary. *Nano Lett.* **14**, 6342-6347 (2014).
7. Orlando, F. *et al.* Epitaxial Growth of Hexagonal Boron Nitride on Ir(111). *J. Phys. Chem. C* **116**, 157-164 (2012).
8. Hu, X., Björkman, T., Lipsanen, H., Sun, L. & Krasheninnikov, A. V. Solubility of boron, carbon, and nitrogen in transition metals: getting insight into trends from first-principles calculations. *J. Phy. Chem. Lett.* **6**, 3263-3268 (2015).
9. Farwick zum Hagen, F. H. *et al.* Structure and growth of hexagonal boron nitride on Ir(111). *ACS Nano* **10**, 11012-11026 (2016).
